# MethScore as a new comprehensive DNA methylation-based value refining the prognosis in acute myeloid leukemia

**DOI:** 10.1186/s13148-024-01625-x

**Published:** 2024-01-22

**Authors:** Šárka Šestáková, Cyril Šálek, Dávid Kundrát, Ela Cerovská, Jan Vydra, Ivana Ježíšková, Adam Folta, Jiří Mayer, Petr Cetkovský, Hana Remešová

**Affiliations:** 1 Institute of Hematology and Blood Transfusion, U Nemocnice 1, 128 00 Prague, Czech Republic; 2 Institute of Clinical and Experimental Hematology, 1st Faculty of Medicine, Charles University, Prague, Czech Republic; 3 Faculty of Science, Charles University, Prague, Czech Republic; 4 Department of Internal Medicine, Hematology and Oncology, University Hospital Brno and Masaryk University, School of Medicine, Brno, Czech Republic

**Keywords:** Acute myeloid leukemia, DNA methylation, NGS, Prognosis

## Abstract

**Background:**

Changes in DNA methylation are common events in the pathogenesis of acute myeloid leukemia (AML) and have been repeatedly reported as associated with prognosis. However, studies integrating these numerous and potentially prognostically relevant DNA methylation changes are lacking. Therefore, we aimed for an overall evaluation of these epigenetic aberrations to provide a comprehensive NGS-based approach of DNA methylation assessment for AML prognostication.

**Results:**

We designed a sequencing panel targeting 239 regions (approx. 573 kb of total size) described in the literature as having a prognostic impact or being associated with AML pathogenesis. Diagnostic whole-blood DNA samples of adult AML patients divided into a training (*n* = 128) and a testing cohort (*n* = 50) were examined. The libraries were prepared using SeqCap Epi Enrichments System (Roche) and sequenced on MiSeq instrument (Illumina). Altogether, 1935 CpGs affecting the survival (*p* < 0.05) were revealed in the training cohort. A summarizing value MethScore was then calculated from these significant CpGs. Patients with lower MethScore had markedly longer overall survival (OS) and event-free survival (EFS) than those with higher MethScore (*p* < 0.001). The predictive ability of MethScore was verified on the independent testing cohort for OS (*p* = 0.01). Moreover, the proof-of-principle validation was performed using the TCGA dataset.

**Conclusions:**

We showed that comprehensive NGS-based approach of DNA methylation assessment revealed a robust epigenetic signature relevant to AML outcome. We called this signature MethScore and showed it might serve as a strong prognostic marker able to refine survival probability of AML patients.

**Supplementary Information:**

The online version contains supplementary material available at 10.1186/s13148-024-01625-x.

## Background

Acute myeloid leukemia (AML) is a hematopoietic malignancy characterized by a substantial heterogeneity in terms of disease prognosis. Despite increasing usage of next-generation sequencing (NGS) allowing sensitive and specific mutational detection, not all AML patients possess genetic markers with a clear predictive role [1]. Refining the AML prognosis is therefore still needed, because their outcome is highly variable [2].

DNA methylation is a well-established and intensively studied epigenetic phenomenon, and its aberration is involved in a variety of different malignancies [3, 4]. In the field of AML research, many investigators focused on DNA methylation and reported its clinical utility for prognostic stratification—reviewed in Yang et al*.* [5]. Importantly, changes in DNA methylation are not only mirroring the underlying genetic variations but have their own indisputable role in AML onset and pathophysiology [6].

Therefore, we introduced a unique and comprehensive approach to assess previously reported prognostic DNA methylation changes at once. We hypothesized that such approach might reveal a robust epigenetic profile with prognostic value. For this purpose, we designed an NGS-based DNA methylation panel comprising of genes previously published as having an impact on AML outcome, altogether with genes generally involved in AML pathogenesis (*WT1* and *HOX* genes), and genes that emerged from our unpublished research to evaluate their collective influence on AML prognosis. List of regions targeted by the methylation sequencing panel (according to the Human GRCh37/hg19 genome assembly) is shown in Additional file 1. This DNA methylation panel was utilized also in our previous DNA methylation validation study [7]. Apart from the current study, we examined selected individual genes and validated their influence on AML prognosis separately.

## Results

### MethScore as a novel epigenetic marker for AML outcome prediction

After the application of Cox univariate regression analysis on the filtered sequencing data (described in Methods section), we found 1935 CpGs significantly affecting OS (*p* < 0.05) in the training cohort (*n* = 128; for basic or detailed molecular and clinical characterization see Table 1 or Additional file 2, respectively). The full list including the positions and average methylation levels of these CpGs is provided in Additional file 3. Out of these presumably prognostically significant CpGs, higher methylation levels indicated better outcome in 1091 CpGs and, on the contrary, worse prognosis in the remaining 844 CpGs. The CpGs were annotated to 222 genes associated mainly with transcription and RNA regulation, DNA binding, and embryonic development. Genes annotated to the most significant CpGs are listed in Table 2. Using these 1935 CpGs, we computed a weighted summary score from methylation levels and Cox regression coefficients for each patient and named it MethScore (details in Methods section). MethScore proved to predict both overall (OS) and event-free survival (EFS) with high accuracy. We divided AML samples from the training cohort according to the median MethScore value, and patients with a lower MethScore had markedly longer OS and EFS than patients with a higher MethScore (logrank test for OS: *p* < 2e−16; for EFS: *p* = 5e−16), see Fig. 1A. MethScore ranged from − 85 to 690 with median 394 and average 380 in the training cohort. To get an overview of acquired MethScore values, we computed the *z*-score and compared it with the average methylation levels (of the 1935 CpGs) and number of mutations, see Fig. 2A. Higher MethScore correlated with lower average methylation (*R* = − 0.56, *p* = 6.2e−12) and weakly with higher number of mutations (*R* = 0.19, *p* = 0.036). We also computed the MethScore for the healthy donors (*n* = 11); the range of values was from 334 to 503, with median 431 and mean 421 (Additional file 4).

**Table 1:** Clinical and molecular characteristics of AML training and testing cohort

Variable	AML training cohort (*n* = 128)	AML testing cohort (*n* = 50)
Age (years)	Median: 55 (range 21–69)	Median: 59 (range 24–75)
Gender (males/females)	68/60	24/26
Leukocytes count [10^9^/l]	Median: 66.5 (range 1–136)	Median: 22.9 (range 0.7–218)
Blasts in bone marrow [%]	Median: 53.4 (0–97.8)	Median: 50 (20–91.8)
*Karyotype (Grimwade 2010)*		
Favorable	9 (7%)	3 (6%)
Intermediate	87 (68%)	38 (76%)
Adverse	30 (23%)	9 (18%)
Not evaluable	2 (2%)	0
*ELN 2017*		
Favorable	33 (26%)	19 (6%)
Intermediate	44 (34%)	18 (76%)
Adverse	41 (32%)	13 (18%)
Not evaluable	10 (8%)	0
*FLT3 status*		
Wild-type	95 (74%)	37 (74%)
Internal tandem duplication	32 (25%)	13 (26%)
Not evaluable	1 (1%)	0
*NPM1* status		
Wild-type	80 (62%)	29 (58%)
Mutated	43 (34%)	21 (42%)
Not evaluable	5 (4%)	0
Complete remission after 1st induction	70 (55%)	34 (68%)

**Table 2:** Top 10 significant CpGs from Cox univariate analysis

Gene	*p* value	Genome position (hg19)
*HOTTIP*	0.000039	chr7: 27244052–27244053
*EZH2* distal promoter	0.000061	chr7: 148581518–148581519
*AC012531.2*	0.000084	chr12: 54412344–54412345
*LTB*	0.00012	chr6: 31549043–31549044
*HOXB7*	0.00013	chr17: 46708857–46708858
*TNF*	0.00013	chr6: 31544960–31544961
*HOTTIP*	0.00013	chr7: 27244051–27244052
*EZH2* distal promoter	0.00013	chr7: 148581941–148581942
*BTBD3*	0.00016	chr20: 11899128–11899129
*EZH2*	0.00016	chr7: 148580658–148580659

*HOTTIP* lncRNA associated with *HOXA* cluster, *EZH2* histone-lysine N-methyltransferase, *AC012531*.2 lncRNA associated with *HOXC* cluster, *LTB* lymphotoxin beta, *HOXB7* homeobox gene, *TNF* tumor necrosis factor, *BTBD3* BTB domain containing protein 3

**﻿Fig. 1:**
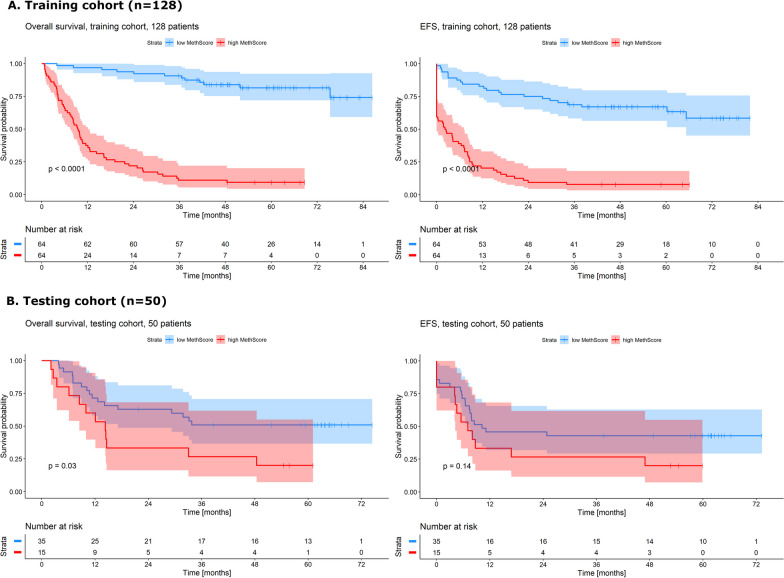
Kaplan–Meier curves with *p*-values of two-sided logrank test comparing both OS and EFS of patients with higher and lower MethScore. **A** In the training cohort (*n* = 128); **B** In the testing cohort (*n* = 50)

**﻿Fig. 2:**
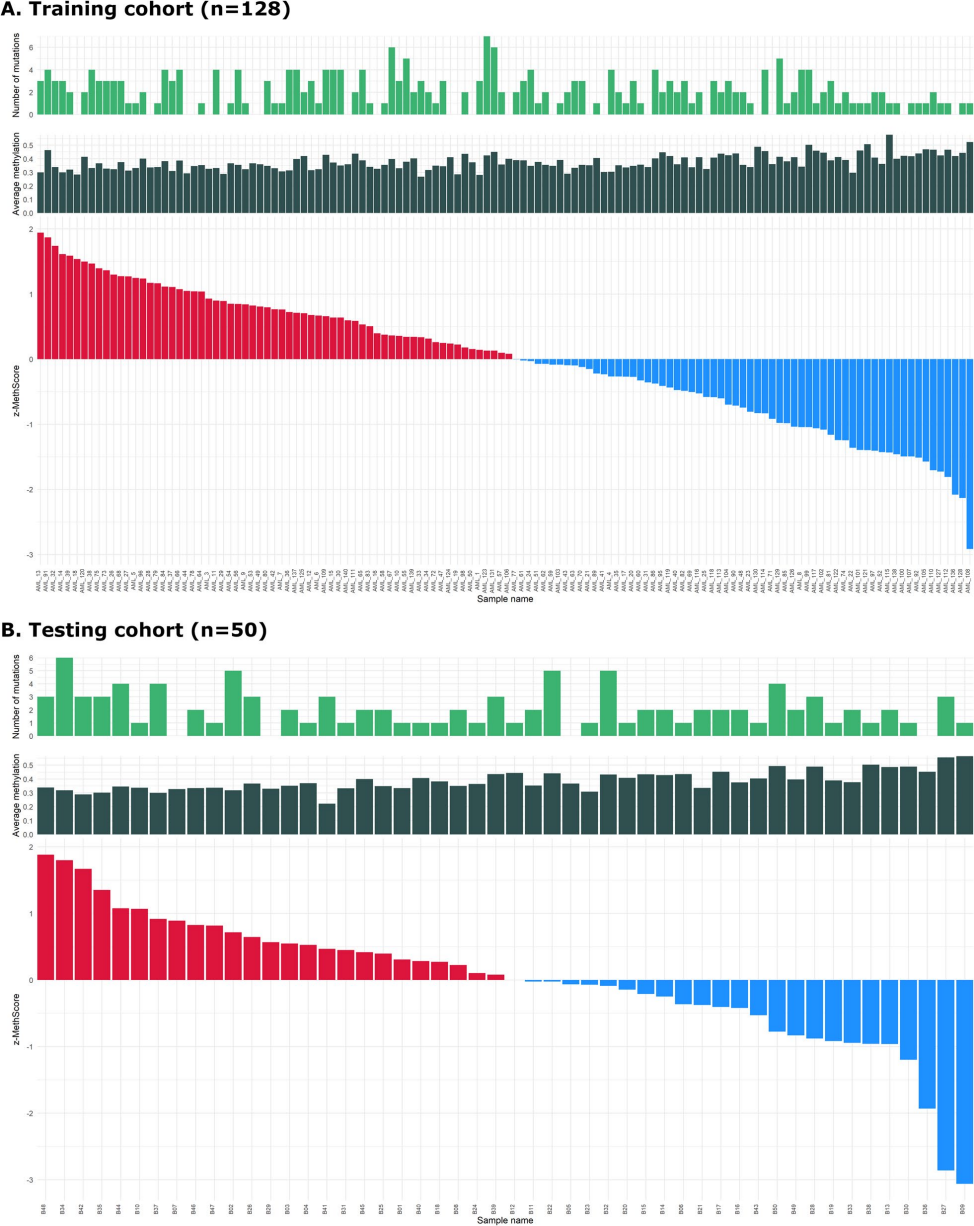
*Z*-score values computed from MethScore together with the average methylation and number of mutations for each patient from **A** the training cohort (*n* = 128); **B** the testing cohort (*n* = 50)

Subsequently, we computed MethScore of the same set of 1935 CpGs for the testing cohort (*n* = 50; for basic or detailed molecular and clinical characterization see Table 1 or Additional file 2, respectively). MethScore values for the testing cohort ranged from − 88 to 584 with median 334 and mean 328. When comparing the survival of AML patients with higher or lower MethScore values (divided by median value of the training cohort), the difference remained significant for OS (logrank test for OS: *p* = 0.03), but not for EFS (logrank test for EFS: *p* = 0.1)—Fig. 1B. Z-score graph together with the average methylation and the number of mutations for the testing cohort are shown in Fig. 2B. Similar to the training cohort data, higher MethScore strongly correlated with lower average methylation (R = − 0.8, *p* = 2.8e−12) and weakly correlated with higher number of mutations (R = 0.27, *p* = 0.061) in the testing cohort.

For both cohorts, we further examined the prognostic relevance of MethScore in multivariate analyses; results are summarized in Figs. 3 and 4, respectively. Firstly, the full model with all tested variables was evaluated. Subsequently, backward stepwise variable selection using the AIC method was implemented to reduce the number of relevant variables. For OS in the training as well as in the testing cohort, MethScore remained among the most significant predictors not only in the full model but also in the reduced one. For EFS in the testing cohort, MethScore did not prove its prognostic capability in the full nor in the reduced model. A comparison of patients with lower and higher MethScore is summarized in Table 3.

**﻿Fig. 3:**
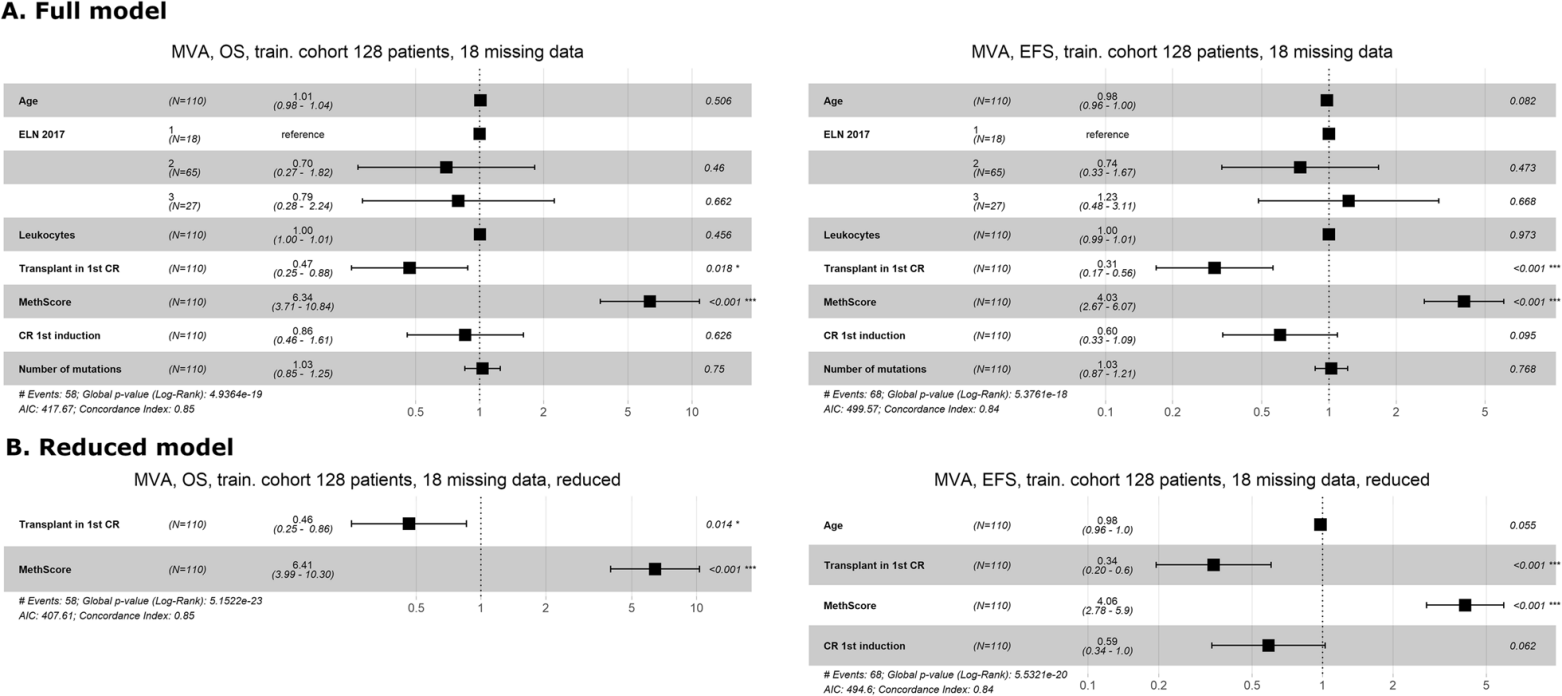
Forest plots from Cox multivariate regression analysis for overall and event-free survival in the training cohort: **A** the full model, **B** the reduced model

**﻿Fig. 4:**
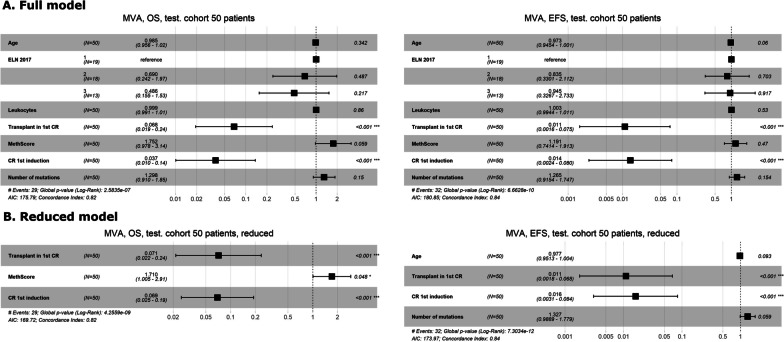
Forest plots from Cox multivariate regression analysis for overall and event-free survival in the testing cohort: **A** the full model, **B** the reduced model

**Table 3:** Comparison of patients with lower (< median) and higher (> median) MethScore

		Training cohort (*n* = 128)			Testing cohort (*n* = 50)		
		Low MethScore (*n* = 64)	High MethScore (*n* = 64)	*p* value	Low MethScore (*n* = 35)	High MethScore (*n* = 15)	*p* value
ELN 2017	Favorable	14	4	**0.004**	13	6	1.000
	Intermediate	36	31		13	5	
	Adverse	8	20		9	4	
	NA	6	9		0	0	
Sex	Male/Female	34/30	34/30	1.000	19/16	5/10	0.224
Transplantation in 1st CR	Yes/No/NA	36/28/0	21/43/0	**0.012**	16/19/0	2/13/0	0.052
Relapse		20/44/0	22/42/0	0.851	15/20/0	8/7/0	0.548
CR after 1st induction		44/19/1	26/37/1	**0.002**	23/12/0	11/4/0	0.746
*FLT3-ITD*	Positive/Negative/NA	14/49/1	18/46/0	0.541	7/28/0	6/9/0	0.170
*DNMT3A* mutation		17/40/7	23/33/8	0.242	5/30/0	5/10/0	0.143
*IDH1/2* mutation		12/45/7	14/42/8	0.660	7/28/0	0/15/0	0.087
*TET2* mutation		4/49/11	7/42/15	0.346	2/33/0	2/13/0	0.574
*ASXL1* mutation		4/49/11	5/44/15	0.735	2/33/0	3/12/0	0.152
*NRAS* mutation		8/45/11	9/40/15	0.184	9/26/0	4/11/0	1.000
*TP53* mutation		0/55/9	12/43/9	** < 0.001**	2/33/0	1/14/0	1.000
*NPM1* mutation		22/40/2	21/40/3	1.000	14/21/0	7/8/0	0.759
*CEBPA* mutation		4/59/1	4/55/5	1.000	2/33/0	1/14/0	1.000
*RUNX1* mutation		4/49/11	5/44/15	0.735	1/34/0	1/14/0	0.514
Number of mutations	Average ± SD/Median	1.7 ± 1.3/1.5	2.3 ± 1.7/2.0	0.099	1.8 ± 1.2/2.0	2.5 ± 1.8/3.0	0.161
Age		45.5 ± 13.4/44.0	55.1 ± 10.0/58.3	** < 0.001**	54.1 ± 14.4/59.0	58.9 ± 12.1/60.0	0.285
Leukocytes		63.6 ± 39.3/61.0	69.4 ± 40.4/70.0	0.400	48.1 ± 59.1/19.9	51.4 ± 63.9/29.0	0.751

*CR* complete remission, *NA* not analyzed, *SD* standard deviation;* p*-values < 0.05 indicated in bold

### Proof-of-principle validation in the TCGA dataset

AML from TCGA study [8] with complete clinically relevant data (*n* = 169, Additional file 5) were split into the training (*n* = 85) and the testing (*n* = 84) cohort. Only CpGs corresponding to genes used in our DNA methylation panel were selected (*n* = 5411) to better reflect our panel data and to reduce number of analyzed CpGs. Subsequently, data were filtered in the same manner as our data. Finally, CpGs associated with survival were determined by univariate Cox regression analysis resulting into 289 significant CpGs (*p* < 0.05) in the TCGA training cohort (*n* = 85). These CpGs are listed in Additional file 6. MethScore calculation was then performed as originally described. MethScore values for the TCGA training cohort ranged from − 399 to − 212 with median − 287 and mean − 290. We separated AML samples from the TCGA training cohort according to the median MethScore value, and patients with lower MethScore had clearly longer OS than patients with higher MethScore (Logrank test for OS: *p* < 5e-04)—see Fig. 5A. The same set of 289 CpGs was utilized for MethScore calculation in the TCGA testing cohort (*n* = 84). MethScore values for the TCGA testing cohort ranged from − 374 to − 225 with median − 268 and mean − 279. Reassuringly, survival difference of AML patients with higher versus lower MethScore values (divided by median value of the TCGA training cohort) remained significant (logrank test for OS: *p* = 0.008)—see Fig. 5B.

**﻿Fig. 5:**
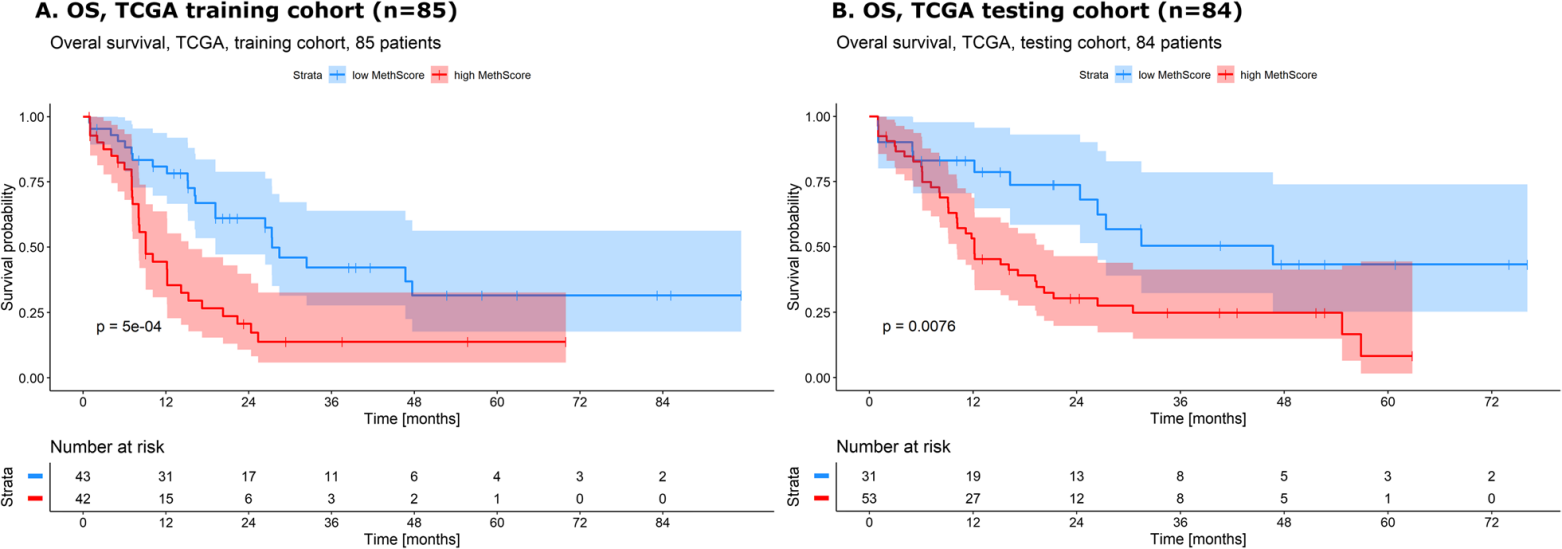
Kaplan–Meier curves with *p*-values of two-sided logrank test comparing OS of AML patients with higher and lower MethScore. **A** in the TCGA training cohort (*n* = 85); **B** in the TCGA testing cohort (*n* = 84)

### CpGs assigned to HOX genes prevail among CpGs associated with AML survival

In the set of 1935 CpGs that were used for the MethScore computation, 636 CpGs (32.9%) were associated with *HOX* genes. Most CpGs belonged to the *HOXA* gene cluster (*n* = 293) and CpGs with lower methylation values indicating better AML outcome prevailed (75%). *HOXB*-associated CpGs were also highly represented (*n* = 148), and there was nearly an equal number of CpGs with prognostically positive lower (51%) and higher (49%) methylation values. The rest of the significant CpGs were assigned to *HOXC* (*n* = 33) and *HOXD* (*n* = 162) gene clusters, and majority of these CpGs (73% and 80%, respectively) were those for which hypermethylation was favorable for AML outcome.

**Fig. 6:**
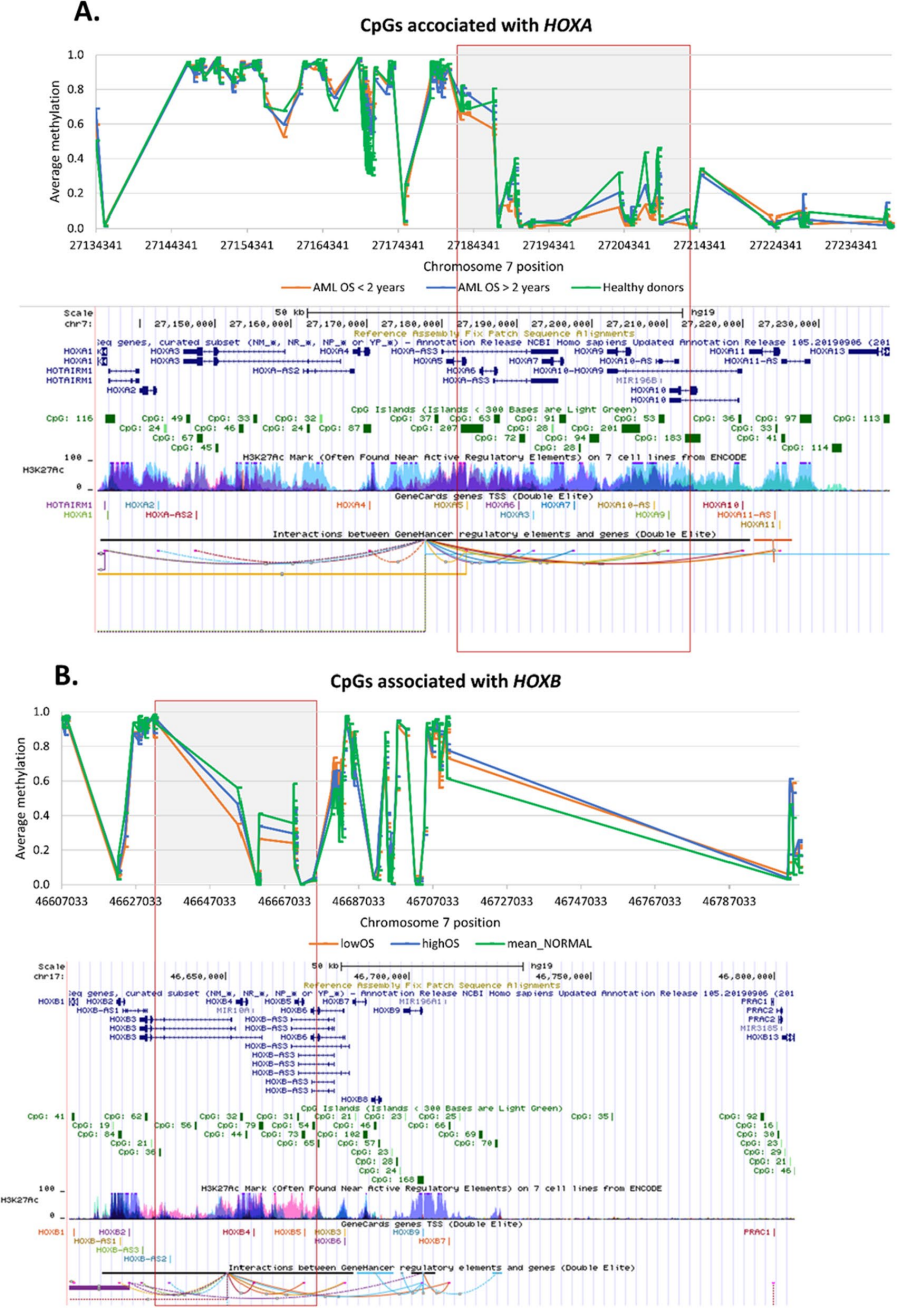
Average methylation levels of CpGs within HOXA/B clusters that were found as significant for patients OS. Values for healthy donors (*n* = 11), AML patients with OS < 2 years (*n* = 54), and AML patients with OS > 2 years (*n* = 74) are shown. The lower half of each image was taken from UCSC Genome Browser, assembly GRCh38/hg19. **A** HOXA gene cluster **B** HOXB gene cluster. Regions with hypomethylation in patients with shorter survival are highlighted

To better understand the observed DNA methylation changes in case of *HOXA* and *HOXB* genes, we plotted the average methylation values of healthy donors and AML samples divided according to their survival, see Fig. 6. There was a distinct region in both *HOX* clusters displaying clear hypomethylation in patients with shorter survival.

## Discussion

A large number of studies already addressed the importance of DNA methylation changes for AML prognosis. Therefore, we designed a custom NGS-based DNA methylation panel comprising of loci/genes from selected studies as well as genes generally connected to AML pathogenesis such as *HOX* genes and *WT1*. Apart from our recent study [7] that validated individual DNA methylation changes and utilized the same AML cohort as well as the DNA methylation sequencing panel, we now wanted to evaluate all potential epigenetic markers comprehensively at once.

We introduced MethScore, a simply computed value that comprehensibly evaluated the prognostic impact of DNA methylation on AML prognosis. As the first step in MethScore assessment, we identified a set of almost two thousand CpGs associated with AML survival. Approximately one-third of these loci was assigned to *HOX* genes, predominantly to *HOXA* and *HOXB* clusters. The indispensable role of homeobox genes in hematopoiesis control is well known, and their impaired expression and aberrant DNA methylation have been implicated as a prognostic marker in AML [9–11]. Overexpression and hypomethylation of *HOXA* genes were reported as a key feature of leukemia stem cells (LSC) signature and validated in several independent AML cohorts in connection with worse survival [12]. Concordantly, we observed hypomethylation within the *HOXA* cluster in AML with shorter survival (Fig. 5A). This region overlaps with 38-kbp region reported as regulatory for *HOXA* locus [13]. Similarly, we noted hypomethylation in a regulatory region of *HOXB* cluster in patients with shorter survival (Fig. 5B). There was also an overlap with locus control region reported for *HOXB* cluster in the study by Spencer et al*.* [13]. This hypomethylation may point to an overexpression of *HOXB* genes that is also well documented as an adverse prognostic factor [14, 15].

Considering the MethScore values, higher MethScore strongly correlated with lower DNA methylation levels and weakly correlated with an increased number of mutations. The higher mutational burden may represent a progressing genome instability that is also characterized by substantial DNA methylation changes [16]. The lower average methylation in patients with higher MethScore and thus adverse outcome probably reflects the previously published discoveries that increased methylation at specific loci may serve as a break preventing AML progression [13], and thus, higher DNA methylation is prognostically more favorable [17]. In the Kaplan–Meier analysis of the training cohort, MethScore had a striking significance for both overall and event-free survival, which was further confirmed in a multivariate Cox regression analysis. The predictive ability of MethScore was also proved in the independent testing cohort for OS, but not for EFS. It must be emphasized that the actual MethScore value can be used for prognostic stratification only if the same experimental setting is kept—essentially, usage of the same input cell type (whole blood), sample preparation and NGS-based DNA methylation analysis. Otherwise, we would recommend to firstly perform MethScore value calculation for a consistent cohort of AML patients in the settings suitable for each laboratory. There might be considerable difference for usage of peripheral blood vs bone marrow, mononuclear cells vs whole blood, sorted blast vs unsorted population. Also, a method of DNA methylation assessment (e.g. NGS vs array) may affect the resulting MethScore value. To provide not only validation of a particular MethScore value that may not be applicable for everyone, we also accomplished a proof-of -principle validation in the publicly available TCGA dataset [8]. Although summarizing DNA methylation value was calculated from lower number of CpGs (*n* = 289) with only minor overlap with 1935 previously determined CpGs (8/1935, see Additional file 6), it justified its applicability for AML prognostication in terms of OS. This assured us of the validity and clinical applicability of MethScore.

## Conclusions

We introduced a novel approach for complex assessment of DNA methylation changes in AML patients. MethScore is based on data measured by NGS, which is a common technique available in nearly all laboratories, and its computation is simple and easy to reproduce. We showed that MethScore may help to improve the risk assessment of AML patients. We believe that after a proper validation, MethScore or some other similarly computed summarizing DNA methylation value may complement the currently used biomarkers and serve as a robust epigenetic marker refining the AML prognosis.

## Methods

### Patients

The training cohort comprised of 128 consecutive non-APL diagnostic AML patients from the Institute of Hematology and Blood Transfusion (Prague, Czech Republic). The testing cohort consisted of 50 consecutive non-APL AML patients from the University Hospital Brno (Brno, Czech Republic). All patients were diagnosed with AML between 2013 and 2016 and were treated with curative intent starting with 3 + 7 induction regimen. Basic clinical characteristics are summarized in Table 1, and detailed information is provided in Additional file 2. The study was approved by the Ethics committees of both participating institutions. All patients and healthy donors provided their informed consent. The research conforms with The Code of Ethics of the World Medical Association.

### DNA methylation sequencing panel

The panel for targeted bisulfite sequencing consisted of 239 loci assigned to 186 genes. The custom probes were made by Roche (Basel, Switzerland). The range of selected regions was 121–35606 bp with an average of 2910 bp and median of 1473 bp. The total size of the panel was 573406 bp. The investigated regions are listed in Additional file 1.

### Targeted bisulfite sequencing

Sequencing libraries consisted of 16–18 samples and were prepared according to the SeqCap Epi protocol (Roche, Basel, Switzerland). Diagnostic whole-blood DNA from AML patients was used. Together with the test cohort, we also analyzed 11 samples from healthy donors. Their DNA was isolated from CD34 + cells harvested from buffy coats by magnetic separation using MicroBeads kit (Miltenyi Biotec, Bergisch Gladbach, Germany). We utilized KAPA HyperPrep Kit (Roche) to prepare the libraries. The DNA (800–1200 ng) was first mixed with the bisulfite-conversion control (unmethylated DNA from phage lambda) provided in the SeqCap Epi Accessory kit (Roche) and then fragmented either via E220 Focused ultrasonicator (Covaris, Woburn, MA, USA) or Bioruptor Pico instrument (Diagenode, Liège, Belgium) to get an average size of 200 bp. For the bisulfite conversion, EZ DNA Methylation Lightning Kit (Zymo Research, Irvine, CA, USA) was used as recommended in the SeqCap Epi protocol. Pooled samples from each library were hybridized for about 68 h with DNA methylation sequencing panel probes. We measured the final concentration of the libraries via qPCR using KAPA Library Quantification Kit (Roche), and the average size of the libraries’ fragments was assessed on 4200 TapeStation System (Agilent Technologies, Santa Clara, CA, USA). Libraries were sequenced on MiSeq instrument (Illumina, San Diego, CA, USA) using the MiSeq Reagent Kit v2 (300-cycles) (Illumina).

### Sequencing data analysis

The quality of raw sequencing data in the form of fastq files was checked using FastQC (version 0.11.8) [18] and MultiQC (version 1.7) [19] software. Reads were then trimmed and filtered using Cutadapt 2.4 (version 2.4) [20], and the quality of reads was checked again. Next, the filtered data were mapped with the mapping software Segemehl (version 0.3.4) [21] to human genome version GRCh37/hg19 with added sequence of Enterobacteria phage lambda NC_001416.1. Mapping statistics were assessed (more than 80% of reads were properly mapped in all samples). The mapped reads in the form of bam files were sorted and indexed by Samtools software (version 1.10). Subsequently, the Haarz tool (version 0.3.4) [21] with enabled "callmethyl" option was used to select methylated positions and create vcf files. These files, containing all methylated positions, were further processed in R software. Positions that corresponded to the lambda phage sequence were separated and used for the bisulfite conversion ratio assessment for each sample (higher than 99% in all samples). Remaining positions were filtered and only CpG positions were left in the data.

### Computation of the MethScore

All computations were performed in R software (version 4.0.0). The initial analysis was done for the training cohort only. Firstly, we filtered out CpGs that were not sequenced in a majority of samples (75%) and 54064 CpGs remained. Next, we selected CpGs where the difference between minimal and maximal methylation values across all samples including healthy donors was more than 20% to evaluate only CpGs that are differentially methylated. We acquired a set of 47622 CpGs. Subsequently, Cox univariate regression analysis of DNA methylation levels of individual CpGs and overall survival was performed. Only those CpGs with significant *p*-value (< 0.05) were selected (*n* = 1935). Next, using a linear combination of methylation levels and Cox regression coefficients of CpGs associated with OS, we counted a weighted summary score and called it MethScore. This computation was adapted from Marcucci et al*.* [22] who used the similar method to count a summarizing score of differential gene expression. The MethScore (MS) for patient *i* was calculated by this equation $MS_{i} = \sum w_{j} \cdot x_{ij}$, where *W*_***j***_ is the Cox regression coefficient for CpG *j* and $x_{ij}$ is a methylation value (range 0–1) for CpG *j* in patient *i*. MethScore for AML samples from the testing cohort was computed via the same equation and for the same subset of CpGs as used in the training cohort. The whole step-by-step procedure and R script is provided within Additional file 7.

### Statistical analyses and definitions

All statistical analyses were performed in R software (version 4.0.0). Overall survival (OS) was defined as time from diagnosis until death of any cause. Event-free survival (EFS) was defined as time from the first complete remission until death or hematological relapse. Kaplan–Meier curves and two-sided logrank test were used to estimate the significance for OS and EFS. Cox regression was performed as uni- or multivariate analyses. For the multivariate analyses (MVA), the input data were corrected to the effect of transplantation by using time-dependent covariate for transplantation. MethScore values used for Cox regression analyses were normalized by z-score method to get a range of values comparable to other variables used in MVA. All multivariate analyses were initially performed with full range of clinical variables. Subsequently, Akaike information criterion (AIC) method was used to reduce the number of tested variables, to keep only relevant ones. For each regression model, the proportional hazards assumption was checked. In the patients’ comparisons, Fisher’s exact test was used to compare the categorical variables, and unpaired two-samples Wilcoxon test (Mann–Whitney test) was used for the continuous variables’ comparison (Table 3). Pearson correlation coefficient (PCC) was utilized for a linear correlation between two sets of data.

### TCGA data analysis

TCGA methylation array data were downloaded from National Cancer Institute portal (https://portal.gdc.cancer.gov/projects/TCGA-LAML). This dataset initially contained 194 AML samples, but it had to be reduced by 25 samples, since information about death of patient and overall survival was not available for these samples. Resulting TCGA dataset therefore contained 169 samples (Additional file 5). Methylation array data were then filtered based on genomic position; only those CpGs were kept, which corresponded with genomic coordinates of our custom sequencing panel (Additional file 1). This filtering resulted in 5411 CpG positions, which were further filtered in the same manner as our panel data. Firstly, only those positions, which had non-NA methylation value in at least 75% of all samples were kept, resulting in table containing 4465 CpG positions. Secondly, only those CpGs where difference between minimum and maximum methylation value across all samples was at least 20% (0.2) were kept. Resulting table contained 3566 CpG positions. Thus, filtered TCGA data were then divided into training (*n* = 85) and testing (*n* = 84) cohort by random sampling. Univariate Cox regression analysis was then performed on all CpG positions left after filtering in TCGA training cohort. Leftover missing data were not addressed, since Cox regression analysis was performed in univariate setting. Out of all 3566 tested CpGs, only those CpGs which were evaluated as significant (*p* < 0.05) were used in subsequent analyses. This resulted in the list of 289 significant CpGs (Additional file 6). MethScore was calculated as previously described for each sample in TCGA training and testing cohort, and subsequent statistical analyses were performed.

### Gene ontology analysis

For the gene ontology analyses, free online programs were used. Bed files containing the positions of selected CpGs were submitted to the online annotation tools GREAT [23] and Enrichr [24]. The gene lists generated in Enrichr from bed files were further submitted to GOrilla tool [25].

### Supplementary Information

**Additional file 1**. List of regions targeted by the DNA methylation sequencing panel (according to the Human GRCh37/hg19 genome assembly).

**Additional file 2**. Detailed molecular and clinical characteristics of 128 AML patients from the training cohort (A) and 50 AML patients from the testing cohort (B) investigated with the DNA methylation sequencing panel.

**Additional file 3**. List of CpGs (n = 1935) with methylation levels significantly affecting the overall survival of training cohort patients.

**Additional file 4**. Characteristics of healthy donors enrolled in the study.

**Additional file 5**. Characteristics of 85 AML patients from the TCGA training cohort (train) and 84 AML patients from the TCGA testing cohort (test).

**Additional file 6**. List of CpGs (n = 289) with methylation levels significantly affecting the overall survival of TCGA training cohort patients.

**Additional file 7**. R script that was used for data analysis—including data filtering and MethScore calculation.

## Data Availability

Raw DNA methylation sequencing data are deposited into GEO repository with the accession number GSE165435 (https://www.ncbi.nlm.nih.gov/geo/query/acc.cgi?acc=GSE165435). The other data supporting the findings of the present study are included in this published article [and its Additional files].
